# Supplementary material for: The Effect of Biological Corrosion on the Hydration Processes of Synthetic Tricalcium Aluminate (C3A)
Source: Materials (Basel). 2023 Mar 10;16(6):2225. doi: 10.3390/ma16062225 (PMC10058350; doi:10.3390/ma16062225)
Supplement: Supplementary file 1 [file materials-16-02225-s001.zip › materials-2234811-SI.pdf]

Supplementary file: TGA analysis

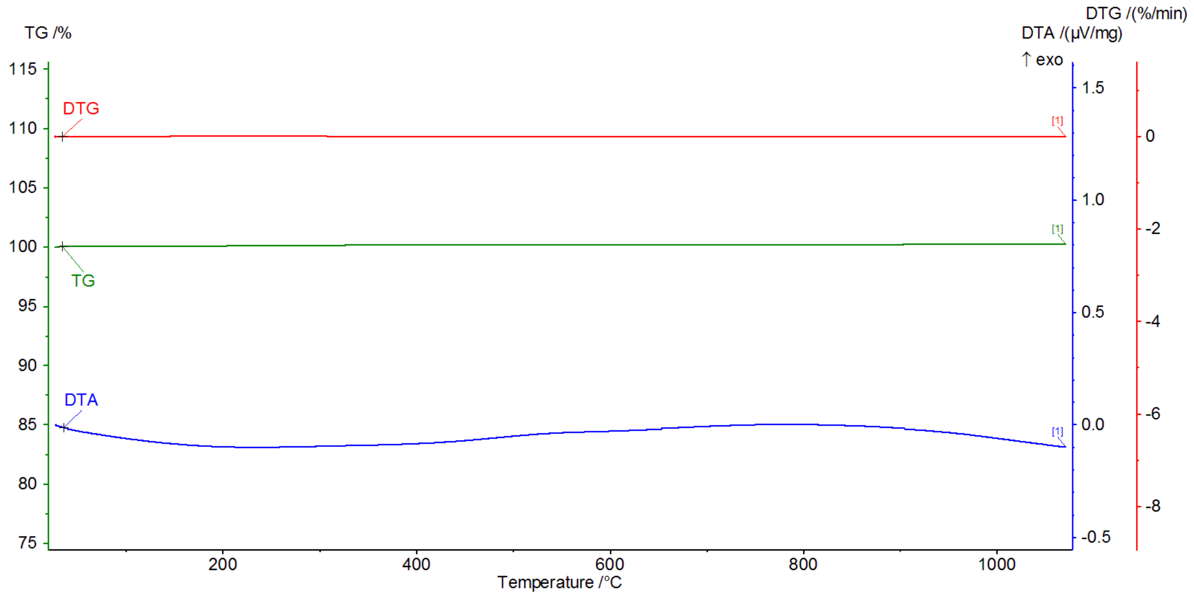

Figure S1. DTA TG DTG thermal curves of dry C_3A (S) as a function of temperature in the range 30-1000°C.

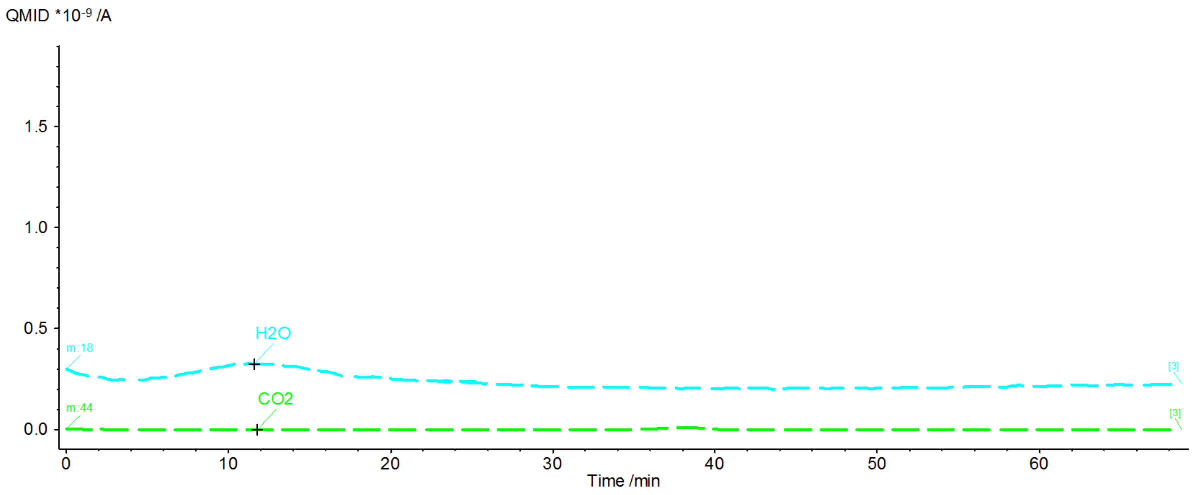

Figure S2. H_2O and CO_2 release curves from dry C_3A as ionic current as a function of temperature.
